# Analysis of the protective effects of γ-aminobutyric acid during fluoride-induced hypothyroidism in male Kunming mice

**DOI:** 10.1080/13880209.2018.1563621

**Published:** 2019-01-24

**Authors:** Haoyue Yang, Ronge Xing, Song Liu, Huahua Yu, Pengcheng Li

**Affiliations:** a Key Laborotory Experimental Marine Biology, Institute of Oceanology, Chinese Academy of Sciences, Qingdao, China;; b Laboratory for Marine Drugs and Bioproducts, Qingdao National Laboratory for Marine Science and Technology, Qingdao, China;; c Center for Ocean Mega-Science, Chinese Academy of Sciences, Qingdao, China

**Keywords:** GABA, thyroid hormone synthesis, myocardial preservation, anti-hypothyroidism

## Abstract

**Context:** Compounds to treat hypothyroidism in the absence of cardiac side effects are urgently required. In this regard, γ-aminobutyric acid (GABA) has gained interest due to its anti-anxiolytic, antihypertensive and antioxidant properties, and reported benefits to the thyroid system.

**Objective:** We investigated the ability of GABA to ameliorate fluoride-induced thyroid injury in mice, and investigated the mechanism(s) associated with GABA-induced protection.

**Materials and methods:** Adult male Kumning mice (*N* = 90) were exposed to NaF (50 mg/kg) for 30 days as a model of hypothyroidism. To evaluate the effects of GABA administration, fluoride-exposed mice received either thyroid tablets, or low (25 mg/kg), medium (50 mg/kg) or high (75 mg/kg) concentrations of pure GABA orally for 14 days groups (*N* = 10 each). The effects of low (50 mg/kg); medium (75 mg/kg) and high (100 mg/kg) concentrations of laboratory-separated GABA were assessed for comparison. Effects on thyroid hormone production, oxidative stress, thyroid function-associated genes, and side-effects during therapy were measured.

**Results:** GABA supplementation in fluoride-exposed mice significantly increased the expression of thyroid TG, TPO, and NIS (*P* < 0.05), significantly improved the thyroid redox state (*P* < 0.05), modulated the expression of thyroid function-associated genes, conferred liver metabolic protection, and prevented changes to myocardial morphology, thus reducing side effects. Both pure and laboratory-separated GABA displayed comparative protective effects.

**Discussion and conclusion:** Our findings support the assertion that GABA exerts therapeutic potential in hypothyroidism. The design and use of human GABA trials to improve therapeutic outcomes in hypothyroidism are now warranted.

## Introduction

The thyroid is one of the most important endocrine glands. It secretes thyroid hormones, including triiodothyronine (T3) and thyroxine (T4), which regulate growth and development, metabolism, reproduction and other important physiological processes. The levels of environmental endocrine disruptors (EEDs) that can influence the endocrine system have increased in recent years. The thyroid is sensitive to EEDs, particularly fluoride (Barberio et al. 2017). Fluorine is widely distributed in nature and is absorbed by the human body through food, water, and air. More than 50 countries have reported endemic chronic fluorosis, which is caused by fluoride in drinking water, coal-burning and brick tea. Fluorosis can also be caused by consuming salts (Chattopadhyay et al. 2011; Li 2011; Huang et al. 2017). Excessive intake of fluoride causes serious thyroid damage. Hillman et al. (1979), Liu (2011), and Desai (1993) reported that fluoride can significantly affect thyroid morphology, resulting in thyromegaly. Furthermore, fluoride inhibits the activity of adenylate cyclase and reduces cyclic adenosine monophosphate (cAMP) formation, resulting in increased thyroid iodine clearance. This leads to a decrease in peripheral T3 and T4 in addition to the inhibition of thyroid hormone transport and function (Juvenal 1978). Moreover, fluorine poisoning in laying hens significantly decreases the level of thyroglobulin (TG) (Liu 2011). Fluoride increases total thyroid stimulating hormone (TSH) and T3, and reduces peripheral T3 and T4 in humans, rats, cattle, laying hens and piglets (Hillman et al. 1979; Yu 1985; Liu et al. 2001; Zhan et al. 2006). Thus, there is an urgent need to identify mechanisms that prevent or ameliorate thyroid dysfunction caused by environmental fluoride pollution.

Most patients with hypothyroidism require thyroid hormone therapy throughout their lives for which synthetic T4 is commonly used (Bartalena et al. 1996). However, long-term thyroid hormone therapy is often associated with side effects, including coronary heart disease, myocardial infarction, heart failure and arrhythmia. Identifying new compounds to treat hypothyroidism and protect the thyroid system without remarkable side effects is required.

γ-Aminobutyric acid (GABA) is an important inhibitory neurotransmitter in the nervous systems of mammals, crustaceans, insects and some parasitic worms. Applications of GABA have gained popularity due to its anti-anxiolytic, antihypertensive, growth-promoting (Siragusa et al. 2007) and antioxidant properties (Inoue et al. 2003; Cho and Chang 2007). To date, the effects of GABA on the thyroid system have been investigated in only a small number of studies. It was demonstrated that the addition of 50–100 mg/kg GABA to the diet of chickens under heat stress significantly increased T3 blood concentrations (Li et al. 2010; Dai et al. 2011; Zhang et al. 2012). Moreover, Xie et al. (2014) demonstrated that treatment with 0.2% and 0.12% GABA normalized plasma TSH levels, improved plasma T4 levels and normalized the morphology of follicles in high-fat-diet-fed mice.

We previously demonstrated that treatment of fluoride-exposed mice with GABA significantly decreased the metabolic toxicity induced by fluoride, restored the microstructural and ultrastructural organization of the thyroid gland, significantly upregulated T4, T3 and TBG levels and increased TSH to appropriate levels in thyroid follicular epithelial cells (Haoyue et al. 2016). However, the mechanism(s) underlying the protective effects of GABA against fluoride-induced thyroid damage, were not investigated.

Here, we further our understanding of the protective effects of GABA on thyroid tissue through investigating its effects on the synthesis of thyroid hormones, oxidative stress in hypothyroidism mice, the expression of thyroid function-associated genes, and the side effects of treatment during hypothyroidism therapy.

## Materials and methods

### Preparation of purified GABA

*Euphausia pacifica* (Parent *Euphausia Dana,* Original name *Euphausia pacifica Hansen, 1911)* was purchased from the Nanshan Aquatic Product Market, Qingdao, China. *Lactobacillus brevis* CGMCC1511, which has a pronounced ability to produce GABA, was purchased from the China Center for the Preservation of Common Microorganisms, Beijing, China. *E. pacifica* solution was mixed with 1% sodium glutamate and used as a culture medium for *L. brevis* for GABA production. Fermentation was performed for 4 days to obtain a GABA rich solution (4.73 g/L). A 732-type cation-exchange resin and high-performance liquid chromatography (HPLC) were used to purify GABA from the fermented solution using a conventional amino acid separation method. The purity of the end-product was 61%. Purified GABA was used to treat fluoride-induced hypothyroidism in subsequent experiments.

### Materials

Thyroid hormone receptor β (TRβ), T4, T3, TG, thyroid peroxidase (TPO), sodium/iodide symporter (NIS), malondialdehyde (MDA), glutathione reductase (GR), ascorbate peroxidase (APX), total cholesterol (TC), low-density lipoprotein (LDL), high-density lipoprotein (HDL) and citrate synthase (CS) test kits were purchased from Nanjing Jiancheng Bioengineering Institute, Nanjing, China. The gene expressions of thyroxine deiodinase DI (Dio1), monocarboxylate transporter 8 (MCT8), retinoid X receptor α (RXRα), members of the extracellular signal-regulated kinase (ERK) 1/2 pathway (mitogen-activated protein kinase 1 [MEK1], ERK1 and ERK2) and tryptophan-rich sensory protein (TSPO) were determined by reverse transcription-polymerase chain reaction (RT-PCR; Shanghai Bioengineering Ltd., Shanghai, China). Thyroid tablets were purchased from Laiyang Biochemical Pharmaceutical Factory (Shandong Province, Laiyang, China). Sodium fluoride (NaF), GABA and other chemicals used in this study were of analytical grade and purchased from the Sigma Reagent Company, Shanghai, China.

### Animals

Adult male Kunming mice (*N* = 90; weight, 18–22 g, 8–10 weeks old) were obtained from the Institute of Drug Inspection of Qingdao. Animals were provided with a standard protein diet (22% protein, Mazuri 5E10) and pure water *ad libitum*. Mice were kept in animal houses maintained at the standard temperature (22–25 °C) and humidity (50%), with an alternating 12 h light/dark cycle. Prior to experiments, the mice were acclimatized to laboratory conditions for 1 week. All experimental procedures were performed in strict accordance with the recommendations of the Guide for the Care and Use of Laboratory Animals of the Institutional Animal Ethical Committee. The study was approved by the Committee on the Ethics of Animal Experiments of the Institute of Oceanology, Chinese Academy of Sciences, Shandong, China. All efforts were taken to minimize animal suffering. The laboratory animal quality certificate code was scxk20140001.

### Experimental design

Mice were divided into two groups of equal average body weights. Experimental mice (*N* = 80) were administered a daily oral dose of 50 mg/kg of NaF for 30 days and served as models of fluoride-induced hypothyroidism. Control mice (*N* = 10) were administered pure water. The oral median lethal dose (LD_50_) of NaF was previously reported to be 191.4–353.3 mg/kg body weight for mice (Hai and Yuxiang 1996). For a sub-chronic toxicity test, the NaF dosage should be 1/20th to 1/5th of the LD_50_ (Haoyue et al. 2016). Previous studies also showed that receiving GABA orally at a daily dose of 1–100 mg/kg had health-promoting effects on endocrine regulation (McCann et al. 1984; Tadashi and Tomoko 2000; Leventhal et al. 2003). Therefore, the daily concentrations of GABA used in this study were set at 50, 75 and 100 mg/kg. To evaluate the effects of GABA administration on hypothyroidism, mice in the fluoride-exposed group were divided into eight groups (*N* = 10 each) and received the following treatment for 14 days: negative control group (NCG, mice receiving only pure water); positive control group (PCG, mice receiving oral thyroid tablets at a daily dose of 50 mg/kg); low concentration of pure GABA (G1, mice receiving pure GABA orally at a daily dose of 50 mg/kg); medium concentration of pure GABA (G2, mice receiving pure GABA orally at a daily dose of 75 mg/kg); high concentration of pure GABA (G3, mice receiving pure GABA orally at a daily dose of 100 mg/kg); low concentration of laboratory-separated GABA (LSG1, mice receiving laboratory-separated GABA orally at a daily dose of 50 mg/kg); medium concentration of laboratory-separated GABA (LSG2, mice receiving laboratory-separated GABA orally at a daily dose of 75 mg/kg) and high concentration of laboratory-separated GABA (LSG3, mice receiving laboratory-separated GABA orally at a daily dose of 100 mg/kg). Administration of pure or laboratory-separated GABA indicated comparable GABA concentrations. Animals were anesthetized using ether prior to blood sampling.

### Thyroid, blood, liver and heart sample collection

After treatment with GABA for 14 days, mice were sacrificed and the thyroid, blood, liver and heart immediately collected. Samples were kept at −80 °C or in 10% neutral-buffered formalin until further analysis.

### Biochemical analyses of the thyroid, blood, liver and heart

Prior to sacrificing the animals, thyroid samples were collected under ether anesthesia. The levels of TG, TPO, NIS, T4, T3, TRβ, TC, LDL, HDL and CS were evaluated using enzyme-linked immunoassay (ELISA). The MDA level was measured based on a thiobarbituric acid (TBA) colorimetric method. The GR and APX levels were measured by spectrophotography. The levels of T4, T3, TRβ, TG, NIS, TPO, AXP, GR, MDA, LDL, HDL, TC and CS were expressed as μg/L, ng/mL, μg/L, μg/L, ng/mL, pg/mL, U/g, U/gprot, μmol/L, mmol/L, mmol/L, mmol/L and U/L, respectively.

### Total mRNA extraction and real-time quantitative PCR

Total RNA of the thyroid was extracted using Trizol (Shanghai Bioengineering Ltd.). Subsequently, mRNA was reverse-transcribed to cDNA using an oligonucleotide dT primer according to the manufacturer’s instructions (Shanghai Bioengineering Ltd.). Platinum Taq polymerase (Shanghai Bioengineering Ltd.) and 4S Red Plus nucleic acid stain (Shanghai Bioengineering Ltd.) were used for RT-PCR. Table 1 summarizes the primer sequences (designed using Primer Premier 5.0 software).

**Table 1.** Primer forward (F) and reverse (R) sequences.

Gene	Primer sequence	PCR amplicon size
M-β-actin-F	GTGCTATGTTGCTCTAGACTTCG	174 bp
M-β-actin-R	ATGCCACAGGATTCCATACC	
M-Dio1-F	CTGTAGGCAAGGTGCTAATGAC	194 bp
M-Dio1-R	AAACTCAGCCCTGTCTTCCA	
M-MCT8-F	TCCATCTTCGGCATCCATAA	88 bp
M-MCT8-R	GGAACTCCACTTGGCGATT	
M-RXRα-F	GCTGGTGTCTAAGATGCGTGA	77 bp
M-RXRα-R	GGTTGAACAGGACAATGGCTC	
M-MEK1-F	TGACGCAGAAGCAGAAGGTG	90 bp
M-MEK1-R	TGAAGACCACTCCACCGTTG	
M-ERK1-F	GGCTTTCTGACGGAGTATGTG	197 bp
M-ERK1-R	GGGGAACCCAAGATACCTAGA	
M-ERK2-F	TGTTCCCAAATGCTGACTCC	93 bp
M-ERK2-R	CCAGAGCCTGTTCAACTTCAAT	
M-TSPO-F	CAGAAACCCTCTTGGCATCC	138 bp
M-TSPO-R	ACCCAAGGGAACCATAGCG	

Dio1: thyroxine deiodinase DI; MCT8: monocarboxylate transporter 8; RXRα: retinoid X receptor α; MEK1: mitogen-activated protein kinase kinase 1; TSPO: tryptophan-rich sensory protein. Gene IDs: M-β-actin (Actb ID: 11461), M-Dio1 (Dio1 ID: 13370), MCT8 (Slc16a2 ID: 20502), M-RXR (Rxra ID: 20181), M-MEK1 (Map2k1 ID: 26395), M-ERK1 (Mapk3 ID: 26417), M-ERK2 (Mapk1 ID: 26413), M-TSPO (Tspo ID: 12257).

### Microstructure of cardiac muscle cells

Mice were sacrificed and the hearts were subsequently removed and fixed overnight in 10% neutral-buffered formalin. Tissues were dehydrated using graded ethanol and embedded in paraffin. Tissue sections (5 μm) were prepared, mounted on slides, and subsequently stained with hematoxylin and eosin. Sections were evaluated using a compound microscope.

### Statistical analysis

Data were expressed as the mean ± standard deviation (SD). Single-group statistical analysis was performed using a Student’s *t*-test and single-factor analysis of variance between multiple groups was performed using the Duncan’s method. A *P* value < 0.05 based on at least three independent experiments was considered statistically significant. SPSS 19.0 software was used for data analysis. According to the Duncan analysis, the letter ‘a’ represents groups with larger mean values. These mean values decreased from ‘a’ to ‘c’. Groups with the same letters were statistically comparable, whilst those with different letters were statistically different (*P* ≤ 0.05).

## Results

### Effects of GABA on blood T4, T3 and TRβ levels

We measured the levels of blood T3 and T4 and liver TRβ after 30 days of exposure to NaF and 14 days of treatment with GABA and thyroid tablets in mice. As shown in Table 2, T4, T3 and THβ levels were significantly lower in the NCG than in the control group (*P* < 0.05). Different doses of GABA increased T4, T3 and THβ to levels significantly higher than those in the NCG (*P* < 0.05). The effect of GABA on increasing T4 was inversely related to the dose; thus, the effects of GABA decreased with increasing dosage. In the PCG, thyroid tablets had positive effects on T4, T3 and THβ levels, but displayed no significant differences to the low-dose GABA groups. The pure GABA and laboratory-separated GABA were associated with similar effects on T4, T3 and TRβ levels (*P* > 0.05). However, the T4 and T3 levels in PCG and GABA treatment groups remained significantly lower than those of the control group (*P* < 0.05).

**Table 2.** Effects of gamma-aminobutyric acid (GABA) on blood thyroxine (T4), triiodothyronine (T3) and thyroid receptor β (TRβ) in hypothyroidic mice.

Group	Control group	NCG	PCG	G1	G2	G3	LSG1	LSG2	LSG3
T4 (μg/L)	92.27 ± 2.02^a^	28.87 ± 1.20^e^	82.59 ± 6.13^b^	71.31 ± 5.73^bc^	60.83 ± 7.01^bcd^	47.43 ± 6.65^d^	74.06 ± 4.00^b^	53.10 ± 8.12^d^	54.48 ± 5.37^d^
T3 (ng/ml)	10.63 ± 0.75^a^	5.91 ± 0.23^c^	7.20 ± 0.48^bc^	8.56 ± 0.86^b^	7.32 ± 0.35^bc^	7.35 ± 0.63^bc^	8.53 ± 0.68^b^	8.79 ± 0.27^b^	7.21 ± 0.28^bc^
TRβ (μg/L)	28.95 ± 0.70^ab^	18.99 ± 1.04^f^	24.76 ± 0.76^cd^	26.57 ± 0.91^bc^	21.01 ± 1.10^ef^	22.14 ± 0.45^de^	30.14 ± 0.43^a^	22.59 ± 0.97^de^	22.38 ± 1.01^de^

‘a’ represents the groups with the largest mean values. The mean values decreased from ‘a’ to ‘e’. Groups marked with different letters are statistically different (*P* ≤ 0.05). NCG: mice administered only pure water for 14 days; PCG: mice administered thyroid tablets at a daily dose of 50 mg/kg for 14 days; G1: mice administered pure GABA at a daily dose of 50 mg/kg for 14 days; G2: mice administered pure GABA at a daily dose of 75 mg/kg for 14 days; G3: mice administered pure GABA at a daily dose of 100 mg/kg for 14 days; LSG1: mice administered laboratory-separated GABA at a daily dose of 50 mg/kg for 14 days; LSG2: mice administered laboratory-separated GABA at a daily dose of 75 mg/kg for 14 days; LSG3: mice administered laboratory-separated GABA at a daily dose of 100 mg/kg for 14 days.

### Effects of GABA on thyroid hormone synthesis

**Figure 1.**
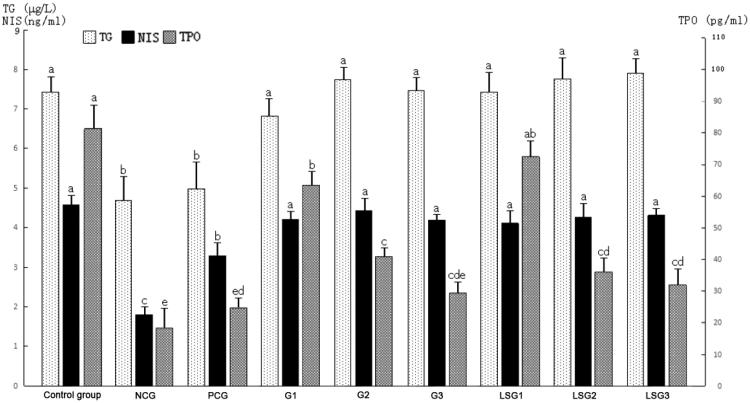
Effects of γ-aminobutyric acid (GABA) on thyroglobulin (TG), sodium/iodide symporter (NIS) and thyroid peroxidase (TPO) in hypothyroidic mice. ‘a’ represents the groups with the largest mean values. The mean values decreased from ‘a’ to ‘e’. Groups marked with different letters are statistically different (*P* ≤ 0.05). NCG: mice administered only pure water for 14 days; PCG: mice administered thyroid tablets at a daily dose of 50 mg/kg for 14 days; G1: mice administered pure GABA at a daily dose of 50 mg/kg for 14 days; G2: mice administered pure GABA at a daily dose of 75 mg/kg for 14 days; G3: mice administered pure GABA at a daily dose of 100 mg/kg for 14 days; LSG1: mice administered laboratory-separated GABA at a daily dose of 50 mg/kg for 14 days; LSG2: mice administered laboratory-separated GABA at a daily dose of 75 mg/kg for 14 days; LSG3: mice administered laboratory-separated GABA at a daily dose of 100 mg/kg for 14 days.

The expression profiles of proteins associated with thyroid hormone synthesis, including TG, TPO and NIS, were measured after 30 days of NaF exposure and 14 days of GABA and thyroid tablet treatment (Figure 1). In NCG, the levels of thyroid TG, TPO and NIS were significantly lower than the control, low-dose pure GABA and laboratory-separated GABA groups. The administration of a low dose of laboratory-separated GABA did not significantly alter the levels of thyroid TG, TPO and NIS compared to the control group (*P* > 0.05). The GABA-induced increase in TG, TPO and NIS was inversely associated with the GABA dose. The effect of low-dose pure GABA and laboratory-separated GABA on the levels of thyroid TG, TPO and NIS were stronger than the thyroid tablets (PCG group). Moreover, the effects of pure and laboratory-separated GABA on TG, NIS and TPO were comparable (*P* > 0.05).

### Effects of GABA on oxidative stress in hypothyroidic mice

**Figure 2.**
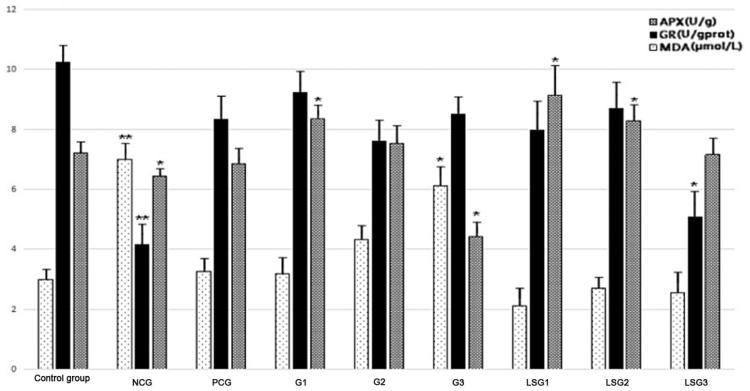
Changes in malondialdehyde (MDA), glutathione reductase (GR) and ascorbate peroxidase (APX) levels after fluoride treatment with or without GABA and liver-protecting tablet administration. Analysis of variance between multiple groups was performed using the least significant difference method. ‘*’ indicates significance at the 0.05 level. ‘**’ indicates significance at the 0.01 level. NCG: mice administered only pure water for 14 days; PCG: mice administered thyroid tablets at a daily dose of 50 mg/kg for 14 days; G1: mice administered pure GABA at a daily dose of 50 mg/kg for 14 days; G2: mice administered pure GABA at a daily dose of 75 mg/kg for 14 days; G3: mice administered pure GABA at a daily dose of 100 mg/kg for 14 days; LSG1: mice administered laboratory-separated GABA at a daily dose of 50 mg/kg for 14 days; LSG2: mice administered laboratory-separated GABA at a daily dose of 75 mg/kg for 14 days; LSG3: mice administered laboratory-separated GABA at a daily dose of 100 mg/kg for 14 days.

Blood MDA, GR and AXP levels were measured after 30 days of NaF exposure and 14 days of GABA and thyroid tablet treatments. The redox indicators of the experimental group are shown in Figure 2. The MDA blood content increased significantly in NCG mice compared with the control group, whilst the levels of GR and APX significantly decreased (*P* < 0.01 and *P* < 0.05, respectively). In PCG, the levels of MDA, GR and APX did not significantly differ from those of the control group (*P* > 0.05). Treatment with GABA at all doses significantly improved the thyroid redox state compared with NCG (*P* < 0.05). However, MDA levels in the high-dose pure GABA group were significantly higher than those of the control group, and GR levels in the high-dose laboratory-separated GABA group were significantly lower than the control group, indicating that the antioxidative effects of GABA decreased with increasing GABA concentrations (Figure 2). These results also indicated differences in antioxidative effects between medium and high concentrations of pure and laboratory-separated GABA. The mechanism(s) underlying these differences require further investigation.

### Effects of GABA on the expression of thyroid function-associated genes

The gene expression of liver Dio1, MCT8, RXRα, thyroid MEK1, ERK1, ERK2 and TSPO were determined after 30 days of NaF exposure and 14 days of GABA and thyroid tablet treatment (Table 3). Relative mRNA levels of Dio1, MCT8, ERK1 and TSPO were significantly upregulated in the NCG compared with the control group (*P* < 0.05) and significantly downregulated in the low-dose GABA groups compared with the NCG (*P* < 0.05). There were no significant differences in the relative mRNA levels of RXRα amongst groups (*P* > 0.05), except for PCG (*P* < 0.05). Relative mRNA levels of MEK1 did not significantly differ amongst groups (*P* > 0.05). Relative mRNA levels of ERK2 modestly increased in NCG compared with the control group (*P* > 0.05). In addition, treatment with GABA at different doses markedly mitigated this increase compared with NCG, although these effects were not significant (*P* > 0.05) except in the high-dose pure GABA group (G3). In the G3 group, the relative mRNA levels of ERK2 were significantly lower than in the NCG (*P* < 0.05). Furthermore, treatment with different doses of GABA had a stronger effect on Dio1, MCT8, ERK1 and TSPO gene expression relative to treatment with thyroid tablets in PCG (*P* < 0.05). The pure and laboratory-separated GABA had similar effects on the regulation of gene expression (*P* > 0.05).

**Table 3.** Effect of GABA on thyroid function-associated genes in hypothyroidic mice.

	Dio1	MCT8	RXRα	MEK1	ERK1	ERK2	TSPO
Control group	30.08 ± 0.31^b^	25.48 ± 0.24^c^	25.67 ± 0.25^bc^	28.62 ± 0.270	25.36 ± 0.17^cd^	25.82 ± 0.50^bc^	23.31 ± 0.40^c^
NCG	32.72 ± 0.27^a^	26.38 ± 0.16^a^	26.17 ± 0.52^ab^	29.00 ± 0.37	26.19 ± 0.11^b^	27.26 ± 0.49^ab^	24.40 ± 0.21^b^
PCG	25.76 ± 0.46^c^	26.44 ± 0.22^a^	26.61 ± 0.11^a^	29.42 ± 0.31	26.67 ± 0.34^a^	28.03 ± 0.35^a^	25.13 ± 0.25^a^
G1	30.96 ± 0.80^b^	25.19 ± 0.21^cd^	25.97 ± 0.75^abc^	29.12 ± 0.68	25.11 ± 0.17^cd^	25.38 ± 0.54^c^	23.66 ± 0.24^c^
G2	30.95 ± 0.31^b^	25.48 ± 0.23^c^	26.05 ± 0.34^ab^	29.16 ± 0.57	25.09 ± 0.17^d^	26.61 ± 0.30^abc^	23.27 ± 0.19^c^
G3	31.44 ± 0.30^ab^	25.91 ± 0.19^b^	26.15 ± 0.16^ab^	29.11 ± 0.32	25.42 ± 0.16^cd^	27.19 ± 0.41^ab^	24.14 ± 0.40^b^
LSG1	30.20 ± 0.26^b^	25.07 ± 0.10^d^	25.54 ± 0.29^bc^	29.94 ± 0.71	25.56 ± 0.21^c^	26.17 ± 0.77^bc^	23.36 ± 0.16^c^
LSG2	30.80 ± 0.25^b^	24.97 ± 0.25^d^	25.27 ± 0.21^c^	29.52 ± 0.86	25.22 ± 0.41^cd^	26.40 ± 0.74^bc^	24.24 ± 0.72^b^
LSG3	31.41 ± 0.67^ab^	24.93 ± 0.15^d^	25.70 ± 0.29^bc^	28.83 ± 0.30	25.09 ± 0.46^d^	25.83 ± 0.66^bc^	24.33 ± 0.64^b^

‘a’ represents the groups with the largest mean values. The mean values decreased from ‘a’ to ‘e’. Groups marked with different letters are statistically different (*P* ≤ 0.05). NCG: mice administered only pure water for 14 days; PCG: mice administered thyroid tablets at a daily dose of 50 mg/kg for 14 days; G1: mice administered pure GABA at a daily dose of 50 mg/kg for 14 days; G2: mice administered pure GABA at a daily dose of 75 mg/kg for 14 days; G3: mice administered pure GABA at a daily dose of 100 mg/kg for 14 days; LSG1: mice administered laboratory-separated GABA at a daily dose of 50 mg/kg for 14 days; LSG2: mice administered laboratory-separated GABA at a daily dose of 75 mg/kg for 14 days; LSG3: mice administered laboratory-separated GABA at a daily dose of 100 mg/kg for 14 days.

### Metabolic enhancement by GABA

The levels of liver LDL, HDL, TC and CS were measured after 30 days of NaF exposure and 14 days of GABA and thyroid tablet treatments. Results are summarized in Table 4. Although none of the treatments altered the levels of LDL (*P* > 0.05), liver TC significantly increased (*P* < 0.05), while HDL-C and CS decreased significantly in NCG compared with the control group (*P* < 0.05). Middle and low doses of GABA significantly promoted liver HDL and CS levels. However, compared with the control group, there were no significant differences (*P* > 0.05). Compared with the NCG, the levels of HDL significantly decreased in PCG (*P* < 0.05), while TC significantly increased (*P* < 0.05); CS levels showed no significant differences (*P* > 0.05). The effect of GABA was superior to that of the thyroid tablets on the HDL and TC levels (*P* < 0.05). The pure and laboratory-separated GABA had similar effects on the regulation of liver metabolism (*P* > 0.05).

**Table 4.** Metabolic enhancement by GABA.

	LDL-C (mmol/L)	HDL-C (mmol/L)	TC (mmol/L)	CS (U/L)
Control group	3.09 ± 0.18	2.04 ± 0.09^a^	6.96 ± 0.76^d^	86.32 ± 5.50^ab^
NCG	2.90 ± 0.13	1.524 ± 0.12^c^	27.67 ± 1.86^a^	59.40 ± 5.51^c^
PCG	2.72 ± 0.17	1.69 ± 0.05^bc^	31.58 ± 2.75^a^	72.25 ± 5.64^bc^
G1	3.13 ± 0.20	2.09 ± 0.08^a^	10.21 ± 1.64^cd^	95.81 ± 45.53^a^
G2	2.78 ± 0.13	1.83 ± 0.14^ab^	12.96 ± 2.61^cd^	75.82 ± 5.40^bc^
G3	2.81 ± 0.04	1.97 ± 0.11^a^	16.00 ± 2.18^bc^	63.58 ± 3.33^c^
LSG1	3.17 ± 0.12	1.92 ± 0.05^a^	11.83 ± 1.46^cd^	97.24 ± 9.21^a^
LSG2	2.98 ± 0.12	1.97 ± 0.07^a^	15.08 ± 2.99^bc^	87.24 ± 6.61^ab^
LSG3	2.69 ± 0.08	1.96 ± 0.07^a^	21.42 ± 0.80^b^	86.42 ± 2.26^ab^

‘a’ represents the groups with the largest mean values. The mean values decreased from ‘a’ to ‘e’. Groups marked with different letters are statistically different (*P* ≤ 0.05). NCG: mice administered only pure water for 14 days; PCG: mice administered thyroid tablets at a daily dose of 50 mg/kg for 14 days; G1: mice administered pure GABA at a daily dose of 50 mg/kg for 14 days; G2: mice administered pure GABA at a daily dose of 75 mg/kg for 14 days; G3: mice administered pure GABA at a daily dose of 100 mg/kg for 14 days; LSG1: mice administered laboratory-separated GABA at a daily dose of 50 mg/kg for 14 days; LSG2: mice administered laboratory-separated GABA at a daily dose of 75 mg/kg for 14 days; LSG3: mice administered laboratory-separated GABA at a daily dose of 100 mg/kg for 14 days.

### The protective effect of GABA on the heart in hypothyroidic mice

**Figure 3.**
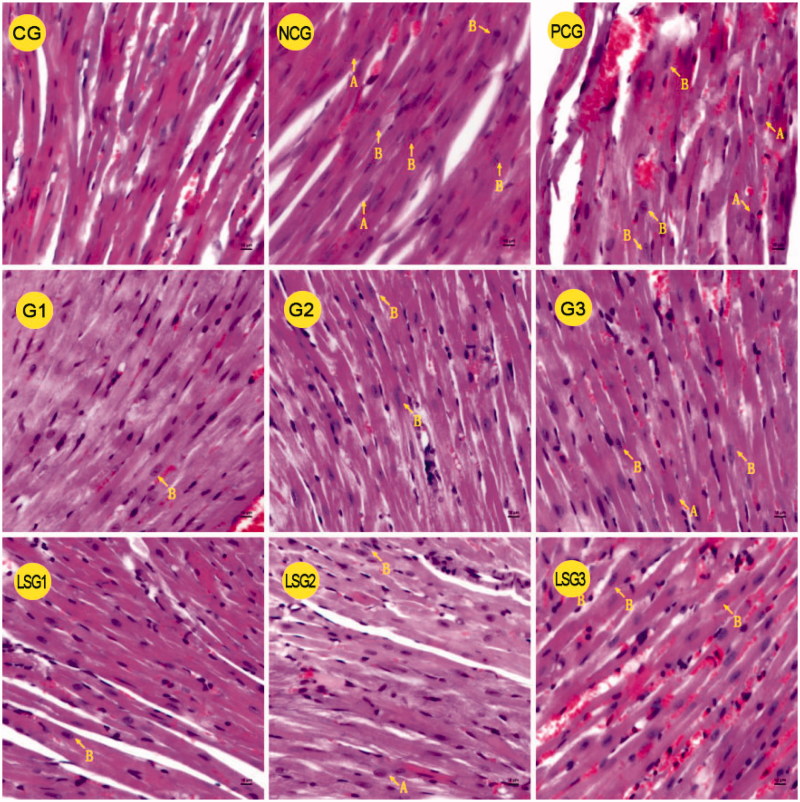
Effects of GABA on the myocardial microstructure of hypothyroidic mice. Arrows marked by the letter ‘A’ depict swelling of the nucleus. Arrows marked by the letter ‘B’ depict karyopyknosis. CG: control group; NCG: mice administered only pure water for 14 days; PCG: mice administered thyroid tablets at a daily dose of 50 mg/kg for 14 days; G1: mice administered pure GABA at a daily dose of 50 mg/kg for 14 days; G2: mice administered pure GABA at a daily dose of 75 mg/kg for 14 days; G3: mice administered pure GABA at a daily dose of 100 mg/kg for 14 days; LSG1: mice administered laboratory-separated GABA at a daily dose of 50 mg/kg for 14 days; LSG2: mice administered laboratory-separated GABA at a daily dose of 75 mg/kg for 14 days; LSG3: mice administered laboratory-separated GABA at a daily dose of 100 mg/kg for 14 days.

Heart HDL levels (Table 5) and myocardial cell microstructure (Figure 3) were analyzed after 30 days of NaF exposure and 14 days of GABA and thyroid tablet treatments. The levels of HDL decreased significantly in NCG compared with the control group (*P* < 0.05). In PCG, the HDL-C levels were significantly higher than the control group (*P* < 0.05). The HDL levels significantly increased in mice treated with different doses of GABA compared with NCG mice (*P* < 0.05), but not control mice (*P* > 0.05). The pure and laboratory-separated GABA had similar effects on the regulation of HDL (*P* > 0.05).

**Table 5.** The protective effects of GABA on the heart of hypothyroidic mice.

Group	Control group	NCG	PCG	G1	G2	G3	LSG1	LSG2	LSG3
HDL-C (mmol/L)	11.42 ± 0.29^b^	7.42 ± 0.12^d^	13.02 ± 0.49^a^	9.11 ± 0.43^c^	10.31 ± 0.44^bc^	11.73 ± 1.12^ab^	10.80 ± 0.16^b^	11.73 ± 0.31^ab^	12.07 ± 0.69^ab^

‘a’ represents the groups with the largest mean values. The mean values decreased from ‘a’ to ‘e’. Groups marked with different letters are statistically different (*P* ≤ 0.05). NCG: mice administered only pure water for 14 days; PCG: mice administered thyroid tablets at a daily dose of 50 mg/kg for 14 days; G1: mice administered pure GABA at a daily dose of 50 mg/kg for 14 days; G2: mice administered pure GABA at a daily dose of 75 mg/kg for 14 days; G3: mice administered pure GABA at a daily dose of 100 mg/kg for 14 daysLSG1, mice administered laboratory-separated GABA at a daily dose of 50 mg/kg for 14 days; LSG2: mice administered laboratory-separated GABA at a daily dose of 75 mg/kg for 14 days; LSG3: mice administered laboratory-separated GABA at a daily dose of 100 mg/kg for 14 days.

In the control group, the arrangement of myocardial fibers was regular and dense; fibers were intact with no fractures, and the nuclear shape was regular with no obvious nuclear condensation (Figure 3(CG)). In NCG and PCG mice, irregular arrangement and rupture of the myocardial fibers were observed, in addition to substantial nuclear pyknosis and swelling (Figures 3(NCG) and 3(PCG)). Compared with NCG and PCG mice, myocardial fibers were arranged neatly, and the swelling and pyknosis of nuclei significantly decreased in GABA-treated mice (Figures 3(G1--G3)--(LSG1--LSG3)). Therefore, the myocardial morphology clearly improved as a result of GABA treatment.

## Discussion

Currently, commercially available pure GABA products are prepared by chemical synthesis, which involves extreme reaction conditions, high energy consumption and poor safety (Siragusa et al. 2007). To avoid these drawbacks, substantial attention has been focused on the use of safe strains for GABA biotransformation. The preparation of GABA by microbial fermentation has the advantages of mild conditions, high content and low production costs (Siragusa et al. 2007). Therefore, we aimed to examine whether GABA isolated from the fermentation broth of aquatic products has the same biological activity as commercially available GABA used at the same concentrations. If these two products exhibit similar activities, then the economical aquatic products or their processing waste can be used as raw material for fermentation to produce GABA preparations, instead of chemical synthesis. With this strategy, it would be possible not only to reduce the cost of producing GABA but to also improve production safety. Nitrogen and trace elements in aquatic products provide sufficient nutrients for microbial growth and do not require supplementation into the culture medium. This simplifies the production of MRS or glucose yeast extract phosphate (GYP) media (currently used in the production of food-grade GABA).

We have previously shown that pure GABA and laboratory-separated GABA (at the same daily dose of 50 mg/kg) exhibited identical protective effects against fluoride-induced hypothyroidism. In particular, they increased blood levels of T4 and T3, protected thyroid follicular epithelial cells from high concentrations of TSH and inhibited apoptosis induced by NaF (Haoyue et al. 2016). However, the mechanism underlying these protective effects remained unclear. Our present findings demonstrate that GABA intake (50 mg/kg per day) significantly increases the synthesis of TG, TPO and NIS, all of which are key to thyroid hormone synthesis, thereby leading to elevated levels of blood T4 and T3.

Previous studies in various model systems reported that oxidative stress and oxygen-derived free radicals play important roles in the pathogenesis of NaF-induced thyrotoxicity. Oxidative stress involving fluoride toxicity causes DNA damage in various cell types (Altuntas et al. 2002), which leads to abnormal metabolism and structural damage. Fluoride poisoning increases blood MDA and decreases blood GR and APX, which are manifestations of oxidative stress. GABA is a powerful antioxidant that protects the thyroid from oxidative stress-mediated cell damage (Inoue et al. 2003; Cho and Chang 2007). In this study, it was shown that a daily dose of 50 mg/kg of GABA over 14 days relieved oxidative stress, decreased MDA content, increased GR and APX activities and restored the antioxidative effects of the thyroid in mice exposed to fluoride.

The involvement of apoptosis in fluorosis is an emerging research topic, and sustained ERK1/2 signaling is intricately linked to apoptosis induction (Chattopadhyay et al. 2011). When normal and malignant cells are stimulated by the external environment, ERK1/2 signaling is activated by phosphorylation, which leads to the translocation of ERK1/2 from the cytoplasm to the nucleus. Activated ERK1/2 induces the transcription of Elk-1, activator protein-1, nuclear factor kappa beta, c-fos and c-jun, which participate in cell differentiation and apoptosis (Goetze et al. 2001; Tresini et al. 2001). In this study, after exposing mice to NaF, ERK1/2 expression increased significantly, suggesting that fluoride mediates apoptosis in thyroid follicular epithelial cells through the sustained activation of ERK1/2. The daily administration of 50 mg/kg GABA over 14 days significantly inhibited ERK1/2, demonstrating that GABA mitigates fluoride-induced apoptosis by inhibiting the activation of ERK1/2. The highly hydrophobic 18-kDa TSPO, located in the mitochondrial outer membrane, is rich in tryptophan and a key factor in mitochondrial apoptosis (Kita et al. 2004; Kita and Furukawa 2008; Nothdurfter et al. 2012; Banati et al. 2014). Indeed, upon stimulation by various chemicals, TSPO becomes upregulated, thus generating mitochondrial reactive oxygen species, activating voltage-dependent anion channels, releasing cytochrome C and activating caspase-9 and caspase-3, which leads to apoptosis (Casellas et al. 2002). Our results showed that the upregulation of TSPO expression induced by NaF was mitigated by treatment with GABA, which blocked mitochondrial apoptosis, thus protecting thyroid follicular epithelial cells.

The thyroid gland is highly susceptible to the effects of fluoride ions (Zeng et al. 2012), which decrease the concentration of T4. Furthermore, the liver is the main site of thyroid hormone metabolism. Therefore, we investigated the effects of GABA on thyroid hormone metabolism in the liver of hypothyroidic mice. It is well established that MCT8 is localized on the surface of the liver where it transfers T4 into hepatocytes. On the other hand, Dio1 is expressed in the liver where it catalyzes the conversion of T4 to T3 and reverse T3, and T3 to diiodothyronine (T2), which is the major source of circulating T3 (Bianco et al. 2002). Therefore, changes in the activity of Dio1 and MCT8 inevitably lead to changes in T4 and T3 levels, disrupting thyroid hormone-directed metabolism. Our results demonstrate that the expression levels of Dio1 and MCT8 increased significantly in NCG compared with the control group, indicating that fluoride improved Dio1 and MCT8 activities, promoted transmembrane deiodination of T4 and decreased T4 serum levels. These findings were consistent with previous studies (Wang et al. 2008; Haoyue et al. 2016). In theory, the increased activity of Dio1 and MCT8 lead to the enhanced conversion of T4 into T3 in the short term, this would increase serum T3 levels. However, our results indicated that fluoride treatment decreased T4 and T3 levels simultaneously. This may have occurred because fluoride ions increase Dio1 activity, which further catalyzed T3 to T2 or monoiodotyrosine, thus decreasing serum T4 and T3 levels in NCG mice. The expression levels of Dio1 and MCT8 decreased after daily treatment with 50 mg/kg of GABA and showed no significant differences in comparison to the control group. This finding indicates that GABA protects normal liver function during the metabolism of thyroid hormones by regulating Dio1 and MCT8 activity.

Thyroid hormones play an important role in balancing glucose and lipid metabolism (Zheng and Ye 2015). Indeed, T3 enhances the levels of glucose-6-phosphatase and CS in the liver, thus promoting the metabolism of sugar. Thyroid hormones can promote lipid degradation, transform cholesterol into cholic acid and excrete it through stools, which can decrease the concentration of serum cholesterol (Williams 1974). Our present results indicate that GABA significantly elevates the level of blood T3 in hypothyroidic mice compared with the control group. GABA treatment can significantly enhance the levels of liver CS and HDL-C and decrease TC levels. However, there were no significant differences in comparison to the control group, which indicates that supplementation with an appropriate dose of GABA can promote glucose metabolism by promoting the synthesis of T3, thus ensuring normal functioning of the thyroid. GABA can also promote lipid metabolism in the liver of hypothyroidic mice by increasing liver HDL-C and decreasing liver TC to levels comparable with those of the control group, only if the thyroid hormone levels are significantly lower than the control group. This indicated that GABA may have a compensatory effect on thyroid hormone function.

Thyroid hormone preparations, particularly T4, are widely used either as replacement doses to correct hypothyroidism or as suppressive doses to abolish TSH secretion in patients with differentiated thyroid carcinoma after total thyroidectomy or with diffuse/nodular nontoxic goiter. To suppress the secretion of TSH, it is necessary to administer slightly supraphysiological doses of T4. Possible adverse effects of this approach include cardiovascular changes (shortening of systolic time intervals, increased frequency of atrial premature beats and, possibly, left ventricular hypertrophy). These can further lead to a range of cardiovascular diseases, such as coronary heart disease, myocardial infarction, heart failure and arrhythmia (Bartalena et al. 1996). Our results demonstrate that, in the process of ameliorating hypothyroidism, GABA had no side effects on the myocardium. In contrast, GABA improved the texture of myocardial fibers and elevated the cardiac levels of HDL-C, an anti-atherosclerosis plasma lipoprotein that protects against coronary heart disease. Furthermore, the levels of HDL-C also increased in PCG, where a level significantly higher than the control group was observed. It has been reported that when HDL-C becomes unusually high, the risk of coronary heart disease increases (Zanoni et al. 2016). This may represent one of the causes of the adverse effects of thyroid hormone replacement therapy.

## Conclusions

Our results demonstrate that both pure and laboratory-separated GABA, at the same GABA concentration, exert comparative protective effects against fluoride-induced hypothyroidism. Under conditions of low T4 levels, GABA treatment maintained the stability of lipid and glucose metabolisms *in vivo*, which has a compensatory effect on the loss of T4. Compared with thyroid tablets, GABA had fewer cardiac side effects and, to some extent, prevented the stress induced by hormone replacement therapy on the heart. These findings further support the assertion that GABA may exert therapeutic potential in hypothyroidism.
